# Supplementary material for: Effects of Rich-Polyphenols Extract of Dendrobium loddigesii on Anti-Diabetic, Anti-Inflammatory, Anti-Oxidant, and Gut Microbiota Modulation in db/db Mice
Source: Molecules. 2018 Dec 7;23(12):3245. doi: 10.3390/molecules23123245 (PMC6320866; doi:10.3390/molecules23123245)
Supplement: Supplementary file 1 [file molecules-23-03245-s001.zip › Supplementary Materials/Table S1. Biological activities of rich-polyphenols extract of D. loddigesii.docx]

**Table S1.** Biological activities of rich-polyphenols extract of *D. loddigesii* (DJP) *in vitro*.

|  | | α-glucosidase | DPPH | NO |
| --- | --- | --- | --- | --- |
|  |  | IC_50_ (μg/mL) | | |
| Sample | DJP | 249.4 | 63.9 | 52.9 |
| Positive control | Vitamin C | n.t^a^ | 5.2 | n.t |
|  | *trans*-resveratrol | 7.7 | n.t | n.t |
|  | L-NG-monomethyl arginine citrate | n.t | n.t | 7.2 |

^a^ n.t: not tested
