# Supplementary figures and images for: Effects of Rich-Polyphenols Extract of Dendrobium loddigesii on Anti-Diabetic, Anti-Inflammatory, Anti-Oxidant, and Gut Microbiota Modulation in db/db Mice
Source: Molecules. 2018 Dec 7;23(12):3245. doi: 10.3390/molecules23123245 (PMC6320866; doi:10.3390/molecules23123245)

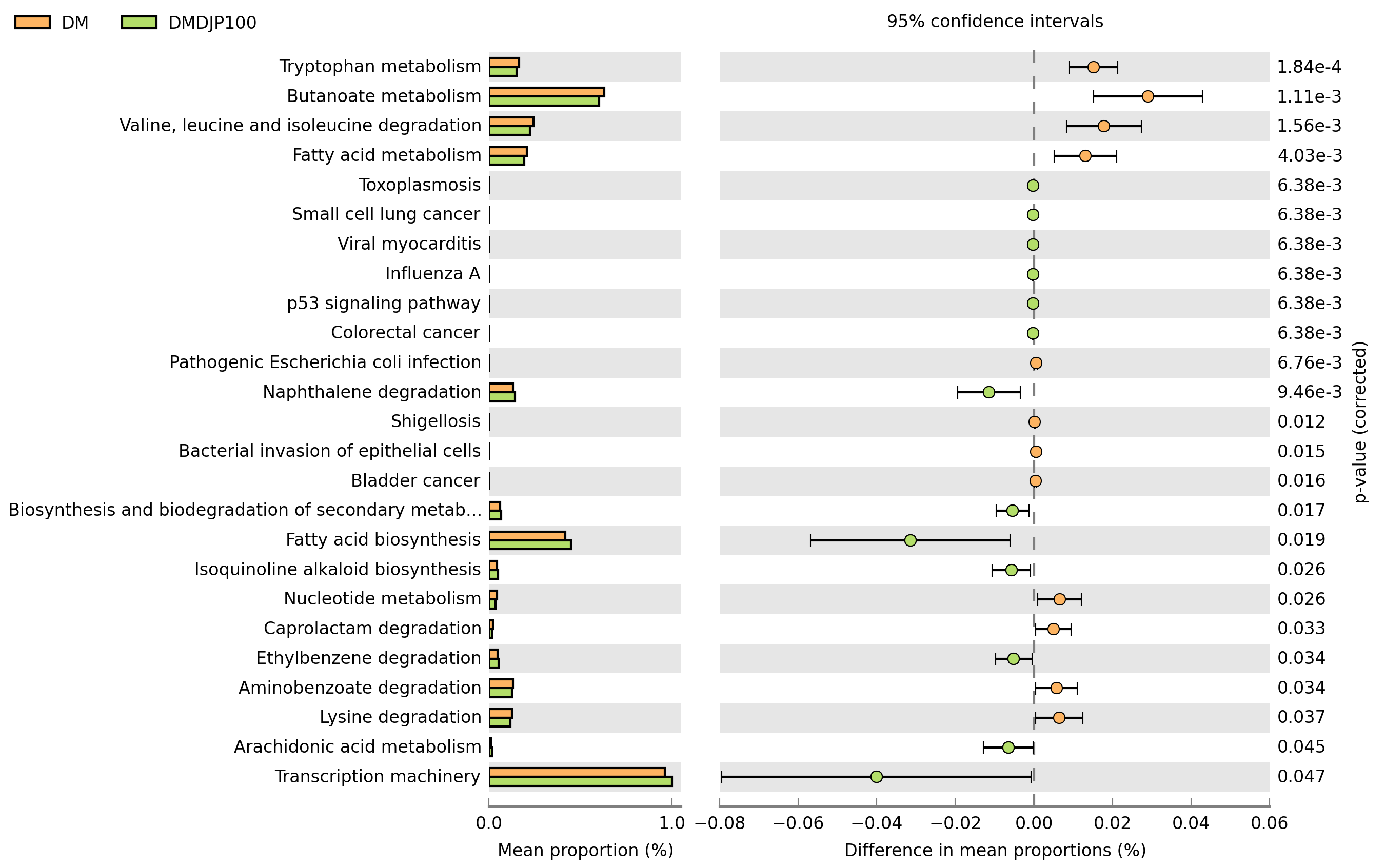

Supplement: Supplementary file 1 [file molecules-23-03245-s001.zip › Supplementary Materials/Figure S2. Metabolic differences between DM and DMDJP100 groups.png]
